# Conservation and Immunogenicity of Novel Antigens in Diverse Isolates of Enterotoxigenic *Escherichia coli*

**DOI:** 10.1371/journal.pntd.0003446

**Published:** 2015-01-28

**Authors:** Qingwei Luo, Firdausi Qadri, Rita Kansal, David A. Rasko, Alaullah Sheikh, James M. Fleckenstein

**Affiliations:** 1 Department of Medicine, Division of Infectious Diseases, Washington University School of Medicine, St. Louis, Missouri, United States of America; 2 International Centre for Diarrhoeal Disease Research, Bangladesh, Mohakhali, Dhaka, Bangladesh; 3 Department of Microbiology, Immunology and Biochemistry, University of Tennessee Health Science Center, Memphis, Tennessee, United States of America; 4 Institute for Genome Sciences, Department of Microbiology and Immunology, University of Maryland School of Medicine, Baltimore, Maryland, United States of America; 5 Molecular Microbiology and Microbial Pathogenesis Program, Division of Biology and Biomedical Sciences, Washington University School of Medicine, St. Louis, Missouri, United States of America; 6 Medicine Service, Veterans Affairs Medical Center, St. Louis, Missouri, United States of America; University of California San Diego School of Medicine, UNITED STATES

## Abstract

**Background:**

Enterotoxigenic *Escherichia coli* (ETEC) are common causes of diarrheal morbidity and mortality in developing countries for which there is currently no vaccine. Heterogeneity in classical ETEC antigens known as colonization factors (CFs) and poor efficacy of toxoid-based approaches to date have impeded development of a broadly protective ETEC vaccine, prompting searches for novel molecular targets.

**Methodology:**

Using a variety of molecular methods, we examined a large collection of ETEC isolates for production of two secreted plasmid-encoded pathotype-specific antigens, the EtpA extracellular adhesin, and EatA, a mucin-degrading serine protease; and two chromosomally-encoded molecules, the YghJ metalloprotease and the EaeH adhesin, that are not specific to the ETEC pathovar, but which have been implicated in ETEC pathogenesis. ELISA assays were also performed on control and convalescent sera to characterize the immune response to these antigens. Finally, mice were immunized with recombinant EtpA (rEtpA), and a protease deficient version of the secreted EatA passenger domain (rEatAp_H134R_) to examine the feasibility of combining these molecules in a subunit vaccine approach.

**Principal Findings:**

EtpA and EatA were secreted by more than half of all ETEC, distributed over diverse phylogenetic lineages belonging to multiple CF groups, and exhibited surprisingly little sequence variation. Both chromosomally-encoded molecules were also identified in a wide variety of ETEC strains and YghJ was secreted by 89% of isolates. Antibodies against both the ETEC pathovar-specific and conserved *E. coli* antigens were present in significantly higher titers in convalescent samples from subjects with ETEC infection than controls suggesting that each of these antigens is produced and recognized during infection. Finally, co-immunization of mice with rEtpA and rEatAp_H134R_ offered significant protection against ETEC infection.

**Conclusions:**

Collectively, these data suggest that novel antigens could significantly complement current approaches and foster improved strategies for development of broadly protective ETEC vaccines.

## Author Summary

Infectious diarrhea is one of the leading causes of death among young children in developing countries, and a major cause of morbidity in all age groups. The enterotoxigenic *Escherichia coli* contribute substantially to this burden of diarrheal illness, and have been a focus of vaccine development efforts for more than forty years following their discovery as a cause of severe diarrheal illness. The heat-labile, and/or heat stable enterotoxins that define ETEC are produced by a diverse population of *Escherichia coli*. This inherent genetic plasticity of *E. coli* has made it difficult to identify antigens specific to ETEC that are highly conserved. Therefore, identification of protective antigens shared by many ETEC strains will likely play an essential role in development of the next iteration of vaccines.

## Introduction

The enterotoxigenic *Escherichia coli* (ETEC) are among the most common causes of infectious diarrhea worldwide. Importantly, ETEC are disproportionately represented in cases of severe diarrheal illness as well as in deaths due to diarrhea among young children in developing countries [1].

These pathogens cause diarrhea by the elaboration and effective delivery of heat-labile and/or heat-stable enterotoxins to intestinal epithelial cells where they stimulate production of cyclic nucleotides ultimately activating the cystic fibrosis transmembrane regulator (CFTR) with resulting net efflux of fluid into the intestinal lumen[2]. Plasmid-encoded colonization factors (CFs), discovered [3] shortly after these organisms were identified as a causative agent of cholera-like diarrheal illness[4–6], are thought to be essential for effective colonization of the small intestine and required for ETEC pathogenesis.

Following early studies suggesting a pivotal role for these structures[7,8], CF antigens have defined the basis for most subsequent ETEC vaccine efforts [9,10]. However, one factor complicating development of a broadly protective vaccine for ETEC has been the general plasticity of *E. coli* genomes[11], and the significant antigenic heterogeneity of the CFs. To date, at least 26 antigenically distinct CF antigens have been described[12]. The lack of appreciable cross-protection afforded by these antigens combined with the complex landscape of CFs portrayed in ETEC molecular epidemiology studies continue to complicate rational CF antigen selection[13].

Antigenic heterogeneity, recent failure of LT-toxoid-based vaccine strategies[14,15], as well as the need to optimize the performance of live-attenuated vaccines currently in clinical trials [16–18] have highlighted the need to identify additional virulence molecules that might be targeted in ETEC vaccines. Recent efforts led to the identification of two loci discovered on the same virulence plasmid of ETEC strain H10407 that encodes the CFA/I colonization factor. These include the *etpBAC* two partner secretion locus responsible for production and export of EtpA[19], a novel adhesin molecule which bridges highly conserved regions of flagellin and the eukaryotic cell surface[20]. Also located on this plasmid is the *eatA* gene that encodes the EatA serine protease autotransporter molecule[21] capable of degrading EtpA[22] as well as MUC2[23], the major gel-forming soluble mucin in the small intestine[24].

Recent immunoproteomic[25] and transcriptomic [26] analyses of H10407 have also highlighted two chromosomally encoded antigens that are not specific to the ETEC pathovar, but which nonetheless appear to be involved in the pathogenesis of these organisms. Conceivably, these molecules, YghJ[27], a secreted mucin-degrading metalloprotease, and EaeH [28], an adhesin, act in concert with colonization factors and other pathovar-specific virulence proteins like EatA and EtpA to promote toxin delivery.

While emerging data suggests that these novel proteins are highly immunogenic[25] and that EtpA and EatA are protective antigens[29–31] in a murine model of ETEC infection, additional data regarding their conservation among ETEC strains are needed to determine their suitability as vaccine targets. Here we demonstrate that these antigens are broadly represented in a diverse collection of ETEC isolates suggesting that they could be employed to augment existing approaches to ETEC vaccine development.

## Materials and Methods

### Bacterial strains and growth conditions

ETEC strains used in this study are detailed in S1 Dataset. All strains were grown at 37° in Cassamino acids yeast extract media[32] (CAYE: 2.0% Casamino Acids, 0.15% yeast extract, 0.25% NaCl, 0.871% K_2_HPO_4_, 0.25% glucose, and 0.1% (v/v) trace salts solution consisting of 5% MgSO_4_, 0.5% MnCl_2_, 0.5% FeCl_3_) from frozen glycerol stocks maintained at −80°C.

### Strain characterization by disease severity and colonization factor type

Strains from the International Centre for Diarrhoeal Disease Research (icddr,b) in Dhaka were selected based on their associated disease severity using modified WHO guidelines as previously outlined[33]. Expression of individual CFs was determined by dot immunoblotting with monoclonal antibodies specific to each respective CFs (CF-MAb) as previously described [34]. Briefly, 2 μl of a PBS suspension containing ∼10^6^ colony forming units of each ETEC strain was dotted onto nitrocellulose, air-dried, blocked with BSA in PBS, followed by detection with CF-MAbs and goat anti-mouse IgG_HRP conjugate. Bound MAbs were then detected with 4-chloro-1-naphthol chromogen and H_2_O_2_.

### Screening for ETEC virulence genes by PCR

We screened a total of 181 ETEC available isolates currently maintained as frozen glycerol stocks in our laboratories. The majority of these strains were collected between 1998 and 2011 in Bangladesh, and were obtained from the icddr,b in Dhaka. Complementing this collection were geographically disparate strains associated with severe diarrheal illness including strains from the Amazon region in Brazil [35], and ThroopD, an isolate from a patient with severe ETEC diarrheal illness who presented in Dallas in the 1970s[36]. Strains encoding *eatA* and *etpA* were identified by PCR using primers directed against conserved regions of these genes as previous described [37]. Briefly, a small amount of frozen glycerol stock from each strain was introduced with a sterile pipette tip into a PCR mixture containing the respective primers and a master mix. Toxin genotypes were confirmed in these isolates using multiplex PCR screening for genes encoding heat-labile (LT), and heat-stable toxins (STp, and STh) as previously described[34]. Primer sequences are listed in S1 Table.

### Immunoblotting for secreted ETEC virulence antigens

To determine production of secreted virulence antigens by different ETEC strains, supernatants from overnight cultures were first precipitated with trichloroacetic acid (TCA) [19] and resuspended in sample buffer before polyacrylamide gel electrophoresis. Western blotting was then performed using polyclonal rabbit antisera against recombinant versions of either EatA[21], EtpA[19], or YghJ[27] that were pre-absorbed against an *E. coli* lysate column (Pierce) and affinity-purified using the antigen immobilized on nitrocellulose membranes as previously described [31,38], followed by detection with affinity-purified secondary goat anti-rabbit-(IgG)-HRP conjugate (Santa Cruz Biotechnology, SC2004).

### Protein sequence comparisons of ETEC pathovar specific antigens

To examine antigenic conservation of EatA among ETEC isolates for which genomic DNA sequences are currently available, BLASTP[39] was used to search GenBank https://www.ncbi.nlm.nih.gov/genbank/ using the full length sequence of the EatA protein from strain H10407 (https://www.ncbi.nlm.nih.gov/protein/AAO17297.1) as the query sequence. To construct alignments of EatA from positive strains, the 1042 residue passenger domain (corresponding to amino acids 57–1098 of EatA from H10407) was compared with EatA of ETEC isolates derived from different phylogenic lineages using a CLUSTAL Omega (release 1.2.0 AndreaGiacomo) [40] algorithm plugin for CLC Main Workbench v6.9.1. A similar approach was used to compare the amino-terminal sequence of EtpA (amino acids 1–600, GenBank accession number AAX13509.2).

### Conservation heat mapping

Virulence protein expression data from the collection of 181 strains under study were included in the analysis. Heat maps were configured using R[41] version 3.1.0 (2014, http://www.R-project.org/) using gplots[42] and RColorBrewer[43] packages installed from http://CRAN.R-project.org using the heatmap2 function within gplots (see S2 Dataset).

### Recombinant protein production

The antigens used in these studies were produced as polyhistidine-tagged recombinant proteins and purified by immobilized metal ion affinity chromatography (IMAC) as previously described[27,29,44,45]. Additional polishing steps including size exclusion or ion exchange chromatography were performed as needed to produce highly purified antigens. Purity of each antigen was assessed by SDS-PAGE followed by sensitive Coomassie Blue staining. Purified recombinant antigens were stored at −80°C.

### Assessment of immune responses to novel ETEC virulence proteins

To quantitfy antibody concentrations directed at novel recombinant antigens, kinetic ELISA was performed on dilutions of plasma samples previously obtained from patients hospitalized at the International Centre for Diarrhoeal Disease Research in Dhaka, Bangladesh (icddr,b) with acute symptomatic ETEC infections. Plasma samples from non-infected adults and children obtained at icddr,b, or specimens obtained from children at Saint Louis Children’s Hospital were used as negative controls. Samples from human volunteer ETEC H10407 challenge studies were kindly provided by Dr. Robert Gormely and Dr. Stephen Savarino of National Naval Medical Center, Bethesda Maryland.

Use of these clinical materials was approved by the Institutional Review Boards of both icddr,b and Washington University School of Medicine. All plasma samples were maintained at 4°C in a humidified chamber prior to use in ELISA. Immune responses to purified recombinant proteins (rYghJ, rEaeH, rEtpA, rEatA_p_) were assessed by kinetic ELISA[46] as previously described [30,47]. Antigen binding to ELISA wells (Corning, Costar 2580) was first optimized to determine the optimal coating concentration and buffer system, using highly antigen-specific polyclonal rabbit antisera to detect binding by ELISA. Purified antigens were then diluted either in 50 mM carbonate buffer (pH 9.6) (rEtpA-myc-His_6_, 1 μg/ml; rEatA_p,_ 10 μg/ml; rYghJ-myc-His_6_, 1 μg/ml); or in phosphate buffered saline (PBS, pH 7.4) (rEaeH-myc-His_6_, 1 μg/ml). ELISA plate wells were coated with 100 μl/well overnight at 4°C, washed with PBS containing 0.05% Tween-20 (PBS-T), and blocked for 1 h at 37°C with 1% BSA in PBS-T. All plasma samples were diluted at 1:4096 in blocking buffer. After incubation for 1 hour at 37°C, plates were washed with PBS-T, and secondary goat anti-human IgG(H+L)-HRP conjugated antibody (Pierce, 31410) was added at a final concentration of 1:10,000. After incubation for 30 minutes at 37°C, plates were washed and developed with TMB microwell peroxidase substrate [3,3’,5,5’-Tetramethylbenzidine] (KPL, 50-76-00). Kinetic absorbance measurements were determined at a wavelength of 650 nm, and acquired at 40 s intervals for 20 minutes using a microplate spectrophotometer (Eon, BioTek). All data were recorded and analyzed using Gen5 software (BioTek) and reported as the Vmax expressed as milliunits/min. Statistical calculations were performed using Prism v4.0c (GraphPad Software), using nonparametric Mann-Whitney (two-tailed) comparisons of data.

### Mouse immunization and challenge studies

These studies were performed in strict accordance with the recommendations in the Guide for the Care and Use of Laboratory Animals of the National Institutes of Health, using an established protocol approved by the Washington University School of Medicine Animal Studies Committee.

Four groups of twelve CD-1 mice were immunized intranasally with either 1 **μ**g of LT (adjuvant only controls), or 1 **μ**g of LT + 15 **μ**g of rEatAp(H134R), or 1 **μ**g of LT + 15 **μ**g of rEtpA, or 1 **μ**g of LT + 15 **μ**g of rEatA(H134R)+15 **μ**g of rEtpA on days 0, 14, 28. On day 40, mice were treated with streptomycin [5 g per liter] in drinking water for 24 hours, followed by drinking water alone for 18 hours. After administration of famotidine to reduce gastric acidity, mice were challenged with 10^6^ cfu of the kanamycin-resistant (*lacZYA*::KmR) strain jf876[48] by oral gavage as previously described[47]. Fecal samples (6 pellets/mouse) were collected on day 42 before oral gavage, re-suspended in buffer (10mM Tris, 100mM NaCl, 0.05% Tween 20, 5mM Sodium Azide, pH 7.4) overnight at 4°C, centrifuged to pellet insoluble material, and recover supernatant for fecal antibody testing (below). Twenty-four hours after infection, mice were sacrificed, sera were collected, and dilutions of saponin small-intestinal lysates were plated onto Luria agar plates containing kanamycin (50 **μ**g/ml).

Murine immune responses to LT, EatA and EtpA were determined using previously described kinetic ELISA. Briefly, ELISA wells were coated with 1 **μ**g/ml GM1, or 10 **μ**g/ml of rEatAp(H134R), or 1 **μ**g/ml rEtpA in carbonate buffer (15 mM Na2CO3, 35 mM NaHCO3, 0.2 g/L NaN3, pH8.6) overnight at 4°C. Wells were washed three times with phosphate-buffered saline containing 0.05% Tween 20 (PBS-T), blocked with 1% bovine serum albumin (BSA) in PBS-T for 1 h at 37°C, and 100 **μ**l of fecal suspensions (undiluted) or sera (diluted 1:100 in PBS-T with 1% BSA) was added per ELISA well and incubated at 37°C for 1 h. Horseradish peroxidase-conjugated secondary antibodies were used and signal detected with TMB (3,3′,5,5′-tetramethylbenzidine)-peroxidase substrate (KPL) substrate.

### Ethics statement

All animal studies were performed under protocols approved by the Animal Studies Committee of Washington University School of Medicine (protocol number 20110246A1). All procedures complied with Public Health Service guidelines, and The Guide for the Care and Use of Laboratory Animals.

All human studies included were performed under a protocol approved by the Institutional Review Board of Washington University School of Medicine (IRB ID# 201110126). All of the human studies here report anonymous analysis of de-identified pre-existing sera previously stored from earlier studies for which no additional consent was obtained.

## Results

### Conservation of ETEC pathogen-specific secreted antigens

Two novel antigens, the EtpA adhesin, and the passenger domain of the EatA serine protease are encoded on the large 92 kilobase virulence plasmid of the prototypical ETEC strain H10407. Both of these secreted proteins[22,30] are required for H10407 to efficiently deliver heat-labile toxin to target epithelial cells. Furthermore, both of these antigens are immunogenic [25], and induce protective immune responses in a murine model of ETEC intestinal colonization[29,31]. To further assess their utility as potential vaccine antigens, we examined a large collection of ETEC strains that were well characterized with respect to associated clinical metadata pertaining to disease severity and which had not undergone repeated serial passage in the laboratory.

**Figure 1:**
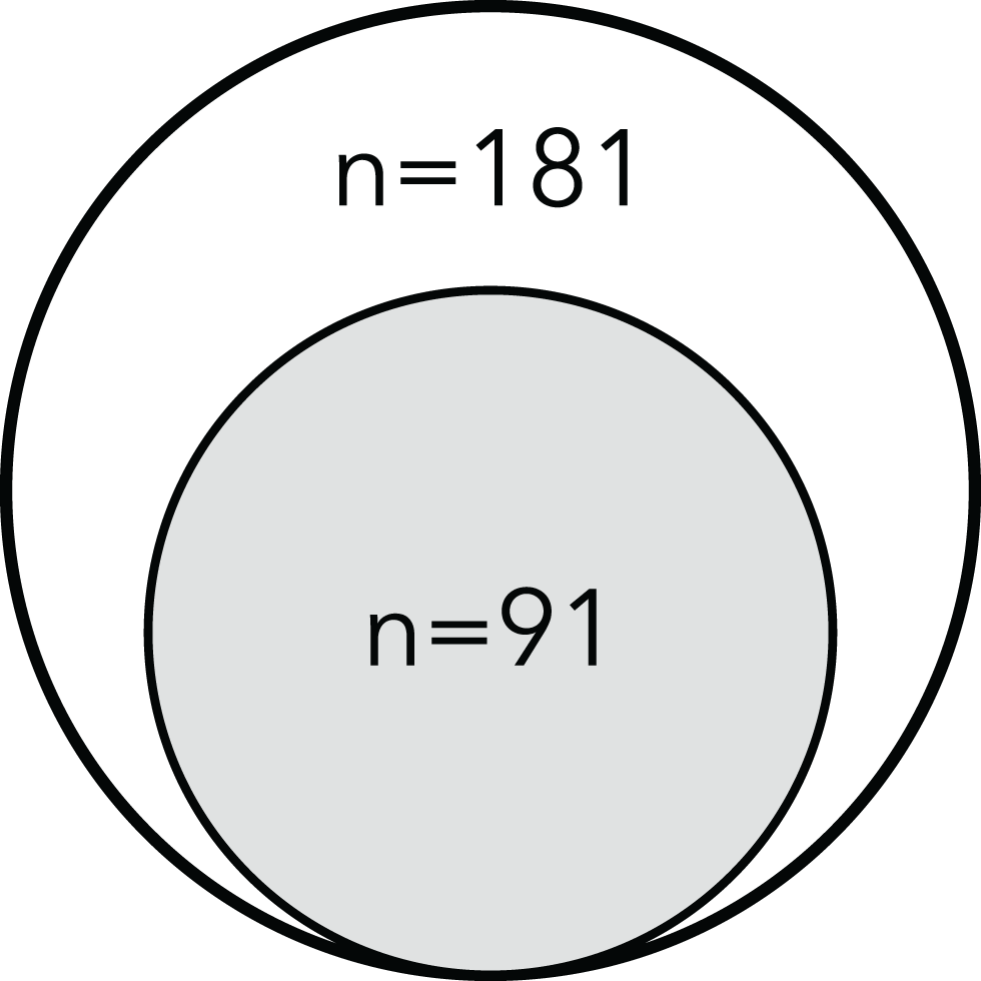
Relationship of strain subsets used in antigen expression studies, and strains with available whole genome sequences. All of the strains in the collection (n = 181) were examined for production of three secreted ETEC virulence proteins EtpA, EatA, and YghJ by immunoblotting of culture supernatants with the respective antibodies. A subset of these strains (n = 91) were recently sequenced at the Genome Sequencing Center for Infectious Diseases (GSCID).

Altogether, we found that these antigens are relatively conserved in the ETEC pathovar, confirming the results of earlier studies that focused on strains from different phylogenies obtained in Guinea Bissau and Chile [37,49]. Of the 181 strains examined in the present study (Fig. 1), we found that more than half of all strains produced EtpA (102/181, 56%) and/or EatA (106/181, 59%) (S1 Dataset), and that more than three quarters of all strains produced at least one of these antigens. Both EtpA and EatA were identified more than twice as frequently as the most commonly identified CF (CS6), which was identified in 22% of strains in this collection (Table 1).

**Table 1:** Distribution of EtpA and EatA among strains expressing different colonization factors.

**CF^1^**	**total (n)**	**EtpA n+ (%)^2^**	**EatA n+ (%)^2^**	**EtpA n+ or EatA n+ (%)^2^**
CFA/I	21	17 (81)	17 (81)	18 (85)
CFA/II^3^				
cs1	14	12 (86)	12 (86)	13 (93)
cs2	10	8 (80)	7 (70)	9 (90)
cs3	25	21 (84)	19 (76)	23 (92)
CFA/IV^3^				
cs4	7	1 (14)	3 (43)	3 (43)
cs5	23	0 (0)	18 (78)	18 (78)
cs6	39	3 (8)	25 (64)	26 (67)
Other CFs				
cs7	16	14 (88)	15 (94)	15 (94)
cs8	2	0 (0)	2 (100)	2 (100)
cs14	14	12 (86)	2 (14)	13 (93)
cs17	10	9 (90)	9 (90)	9 (90)
cs21	17	14 (82)	14 (82)	16 (94)
nd	36	16 (44)	7 (19)	18 (50)

^1^expression as determined with corresponding monoclonal antibody

^2^refers to strains expressing proteins as determined by immunoblotting nd: not detected with any of the above monoclonal antibodies

^3^(CFA/II pili are typically comprised of CS1 or CS2 in combination with CS3; CFA/IV pili may be formed by CS4 or CS5 in combination with CS6 or CS6 alone.)

### Relationship of plasmid-encoded virulence loci to colonization factor antigens

**Figure 2:**
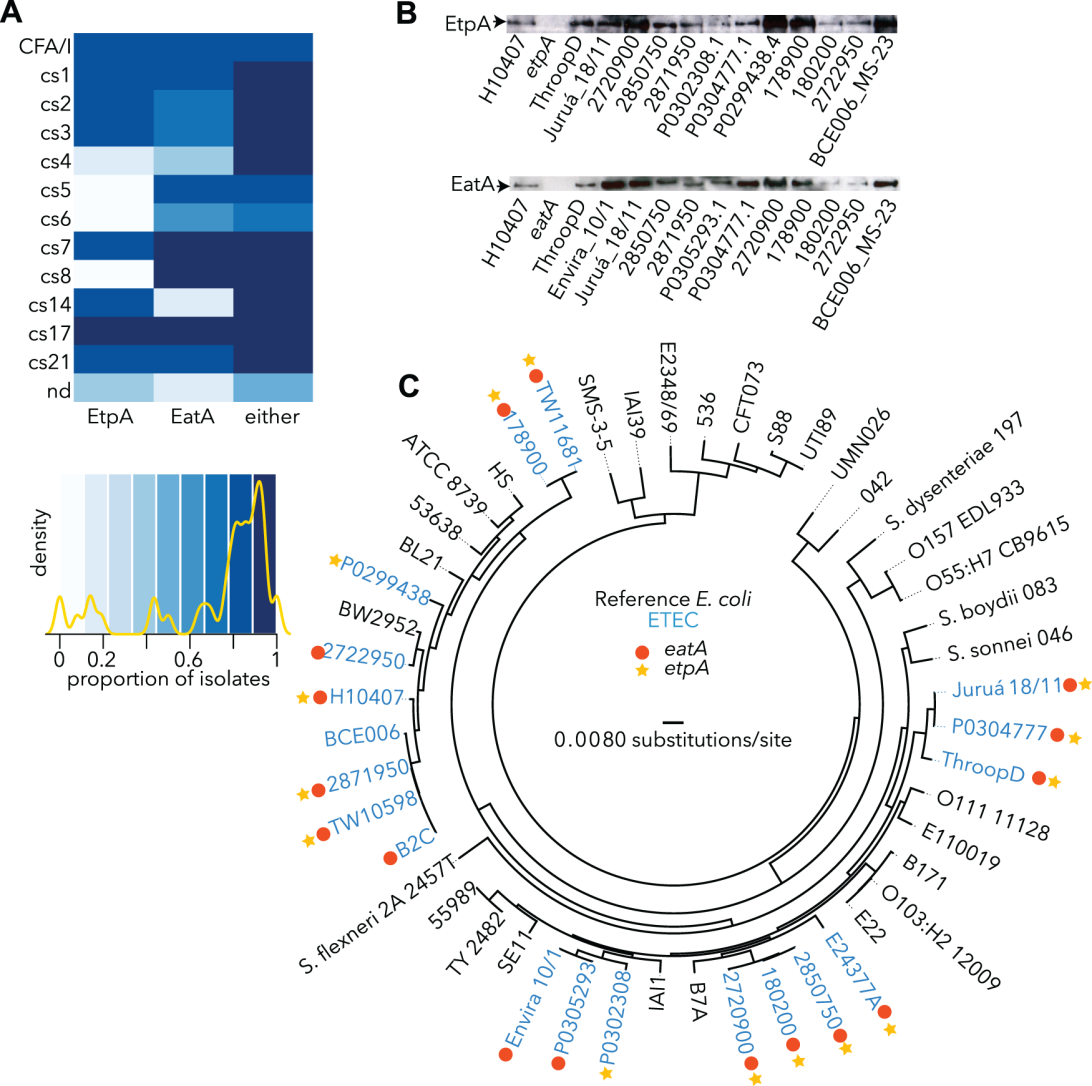
Conservation of novel pathotype-specific antigens EtpA and EatA among phylogenically distinct strains expressing different colonization factors. a. Heatmap of EtpA and EatA showing the proportion of strains positive for expression of these antigens among different CF groups. CF antigen designation is shown at left of the heatmap. nd = no CF antigen detected. Below is the heatmap key depicting colors associated with each degree of antigen positivity. Density line in yellow depicts the relative number of map features assigned at each proportion. Primary data used to construct the heatmap can be found in S2 Dataset. b. Imunoblot detection of EtpA and EatA expression among strains from different phylogenies. The upper immunoblot demonstrates EtpA production in the prototype H10407 strain, ThroopD isolated in Dallas, TX in 1975, the Juruá_18/11 (Amazon, 1998), and phylogentically dispersed strains from icddr,b. The *etpA* mutant is included as a negative control. The lower blot demonstrates EatA production by H10407, phylogenically distributed strains from icddr,b and Envira_10/1, an additional isolate from cholera-like outbreaks in the Amazon. The *eatA* mutant is included as a negative control. c. Phlyogram showing the phylogenetic distribution of selected ETEC strains (designations in blue) and reference E. coli strains (designations in black). Red circles and gold stars represent eatA+, and etpA+ strains, respectively.

Importantly, although the genes encoding the *etpBAC* secretion system[19] and the EatA autotransporter[21] were initially discovered on the same large virulence plasmid of H10407, which also encodes the colonization factor (CF) CFA/I, we found that these loci were not restricted to strains expressing this particular CF, but were widely distributed among the different CFs, and were also present in strains for which no CF could be identified (Fig. 2A). Indeed, half of the strains for which no CF could be identified expressed either EtpA or EatA, suggesting that these antigens could complement existing vaccination strategies centered on CFs. As expected by the association with multiple CFs, we also found that EtpA and EatA were secreted by strains from multiple phylogenic lineages (Fig. 2B, C). Interestingly, however we found a negative association between the *etpBAC* locus and strains expressing CFA/IV antigens [50,51] including CS5 in that none of the 23 strains possessing CS5 fimbriae secreted the EtpA adhesin. Similarly, among strains expressing CS6, which is frequently co-expressed with CS5, only a minority secreted EtpA. These data are also consistent with our earlier observation that the prototype B7A strain, which also expresses CS6, lacks the *etpBAC* locus and does not secrete EtpA[19].

Interestingly, both the *eatA* and *etpBAC* loci were originally identified in ETEC strain H10407, originally isolated from an adult with severe, cholera-like illness in Bangladesh[52]. As has been noted previously, this strain also causes more severe illness in human clinical challenge studies relative to other strains like B7A that lack these loci[53]. Because we had clinical metadata pertaining to disease severity for all of the strains in our collection, we questioned whether the production of either of these antigens was associated with strains isolated from more severe forms of infection. However, we did not find any clear association between either of these putative virulence loci and clinical outcome (S1 Dataset).

### Conservation of chromosomally-encoded antigens

We also examined the conservation of two chromosomally-encoded antigens which are not specific to the ETEC pathovar, but have recently been shown to play a role in virulence. The *eaeH* gene was originally identified on the chromosome of ETEC strain H10407 by subtractive hybridization with *E. coli* MG1655[54], is transcriptionally activated by cell contact [26], and under these conditions EaeH is produced by a diverse group of strains belonging to different phylogenies[28]. Using the EaeH peptide sequence from H10407 (GenBank accession AAZ57201), BLASTP searches of recently sequenced ETEC strains from Bangladesh and elsewhere (http://gscid.igs.umaryland.edu/wp.php?wp=comparative_genome_analysis_of_enterotoxigenic_e._coli_isolates_from_infections_of_different_clinical_severity) also revealed that the *eaeH* gene was present in 63 out of 91 distinct isolates (69%) (S1 Dataset). BLASTP searches of these data for another chromosomally encoded molecule, YghJ, a type II secretion system effector[55] recently shown to be involved in mucin degradation and toxin delivery[27] demonstrated that the *yghJ* gene was present on the chromosomes in 83 of 91 (91%) isolates. Similarly, we identified the YghJ protein in a majority (161/181, 89%) of ETEC culture supernatants (S1 Dataset). This antigen was produced across ETEC strains expressing multiple CF types including 31/36 strains that were CF-negative by monoclonal antibody screening.

### EatA and EtpA sequence conservation

Ideally, putative vaccine targets should be specific to the pathovar under study or restricted to pathogenic isolates, but not subject to significant antigenic variation. Therefore to further examine the potential utility of two ETEC pathovar specific antigens, EtpA and EatA, as vaccine candidates, we used recently obtained DNA sequence information from multiple ETEC genomes belonging to different phylogenies and from temporally and geographically disparate sources to compare the predicted amino acid sequences of these proteins.

For the prototype EatA molecule, first described in ETEC H10407[21], the 1042 residue region from amino acids 57–1098 is predicted for the secreted passenger domain that contains the serine protease catalytic triad[21] as well as protective epitopes[23]. We therefore compared this region of the molecule to those derived from the recently released genome sequences of multiple ETEC strains. Altogether, we found that the sequence of the EatA passenger domain (EatA_p_) was very highly conserved across strains, and exhibited between 95–100% identity to the prototype H10407 Eat_p_ (Table 2). Likewise, the predicted serine protease catalytic motif formed by the histidine, aspartic acid and serine residues at positions 134, 162, and 267, respectively were universally conserved within the passenger domains of these proteins (S1 Fig.). Similarly, the predicted amino acid sequences of the secreted EtpA adhesin molecules from multiple strains exhibited between 94 and 100% identity to the H10407 prototype antigen (Table 3, S2 Fig.).

**Table 2:** EatA sequence conservation in geographically, temporally, and phylogenically disparate isolates.

**strain**	**accession**	**origin**	**isolation reported**	**phenotype**	**identity^1^ (%)**	**Similarity (%)**	**ref.**
H10407	Q84GK0.1	Bangladesh	1973	cholera-like	-	-	[52]
Throop D	**^2^** EMW91712.1	Dallas, TX	1976	cholera-like	98	99	[36]
Envira 10/1	**^2^** EMX71514.1	Amazon, Brazil	1998	cholera-like	97	98	[35]
Juruá 18/11	**^2^** EMX58104.1	Amazon, Brazil	1998	cholera-like	96	98	[35]
2850750	^2^ EMW01116.1	Bangladesh	2008	cholera-like	96	97	^5^
2871950	**^2^** EMV49671.1	Bangladesh	2008	cholera-like	96	98	^5^
P0305293.1	**^2^** EMZ82436.1	Bangladesh	2011	cholera-like	97	98	^5^
P0304777.1	**^2^** EMX02137.1	Bangladesh	2011	cholera-like	96	97	^5^
2720900	**^2^** EMX91421.1	Bangladesh	2007	cholera-like	100	100	^5^
178900	**^2^** ENA71292.1	Bangladesh	2010	cholera-like	98	99	^5^
180200	**^2^** ENA61350.1	Bangladesh	2010	cholera-like	96	98	^5^
2722950	**^2^** EMZ73955.1	Bangladesh	2007	mild-self-limited	98	99	^5^
tw10598	^3^ AELA00000000.1	Guinea-Bissau	1996	diarrhea^4^	96	98	[49]
tw10722	^3^ AELB00000000.1	Guinea-Bissau	1996	diarrhea^4^	95	98	[49]
tw10828	^3^ AELC00000000.1	Guinea-Bissau	1996	diarrhea^4^	95	97	[49]
tw11681	^3^ AELD00000000.1	Guinea-Bissau	1996	diarrhea^4^	98	99	[49]
B2C	ETS27975.1	Vietnam	1971	diarrhea^4^	96	98	[62]
E24377A	YP_001451588.1	Egypt	1980s	diarrhea^4^	96	97	[63]

^1^based on BLAST-P searches against 1042 residues of predicted passenger domain of H10407.

**^2^**sequenced at GSCID (http://gscid.igs.umaryland.edu/wp.php?wp=comparative_genome_analysis_of_enterotoxigenic_e._coli_isolates_from_infections_of_different_clinical_severity)

^3^open reading frames corresponding to the *eatA* gene were first assembled from whole genome shotgun sequence contigs for these draft genomes; BLASTP for these homologues was conducted using CLC Main Workbench v6.9.1 and local database of predicted protein sequences derived from translation of assembled contigs from the respective sequencing projects.

^4^(severity unknown)

**^5^**this study

**Table 3:** EtpA sequence conservation in geographically, temporally, and phylogenically disparate isolates.

**strain**	**accession**	**origin**	**isolation reported**	**phenotype**	**identity^1^ (%)**	**Similarity (%)**	**ref.**
H10407	AAX13509.2	Bangladesh	1973	cholera-like	-	-	[52]
Throop D	^2^ EMW91721.1	Dallas, TX	1976	cholera-like	97	97	[36]
Juruá 18/11	^2^ EMX66921.1	Amazon, Brazil	1998	cholera-like	94	95	[35]
2720900	^2^ EMX81000.1	Bangladesh	2007	cholera-like	100	100	**^5^**
2850750	^2^ EMW11804.1	Bangladesh	2008	cholera-like	94	95	**^5^**
2871950	^2^ EMV50451.1	Bangladesh	2011	cholera-like	95	95	**^5^**
p0302308.1	^2^ EMX03881.1	Bangladesh	2011	cholera-like	98	98	**^5^**
p0304777.1	^2^ EMX02126.1	Bangladesh	2011	cholera-like	99	98	**^5^**
p0299438.4	^2^ ENB94816.1	Bangladesh	2011	cholera-like	99	99	**^5^**
178900	^2^ ENA69446.1	Bangladesh	2010	cholera-like	97	97	**^5^**
180200	^2^ ENA71844.1	Bangladesh	2010	cholera-like	94	95	**^5^**
1392/75	YP_003717617.1	Hong Kong	-	diarrhea^4^	95	96	[61]
tw11681	^3^ AELD00000000.1	Guinea-Bissau	1996	diarrhea^4^	96	97	[49]
tw14425	^3^ AELE00000000.1	Guinea-Bissau	1996	diarrhea^4^	99	100	[49]
tw10828	^3^ AELC00000000.1	Guinea-Bissau	1996	diarrhea^4^	94	96	[49]
tw10598	^3^ AELA00000000.1	Guinea-Bissau	1996	diarrhea^4^	95	96	[49]
E24377A	^3^ NC_009786.1	Egypt	1980s	diarrhea^4^	95	96	[63]

^1^identity and similarity percentages reflect BLASTP comparisons of the first 600 residues of the EtpA sequence.

**^2^**sequenced at GSCID (http://gscid.igs.umaryland.edu/wp.php?wp=comparative_genome_analysis_of_enterotoxigenic_e._coli_isolates_from_infections_of_different_clinical_severity)

^3^open reading frames corresponding to the *etpA* gene were first assembled from whole genome shotgun sequence contigs for these draft genomes; BLASTP for these homologues was conducted using CLC Main Workbench v6.9.1 and local database of predicted protein sequences derived from translation of assembled contigs from the respective sequencing projects.

^4^(severity unknown)

**^5^**this study

Despite the fact that the comparator strains included here spanned isolates collected over nearly 40 years, belonging to different phylogenies and that strains originated in diverse locations in Asia, Africa and the Americas, both proteins appear to exhibit remarkably little antigenic variation. Likewise, in analysis of the genomes of strains isolated recently within Bangladesh both proteins demonstrated similar degrees of sequence conservation (S1–S2 Fig.).

### Immunogenicity of novel virulence antigens

Earlier immunoproteomic studies suggested that a variety of conserved *E. coli* proteins as well as ETEC pathovar specific proteins are recognized during the course of experimental infections in mice, and these responses parallel those observed using pooled convalescent sera from ETEC patients[25]. To further characterize the immune response to novel antigens we focused on four proteins that have recently been shown to play a role in ETEC pathogenesis, including two plasmid-encoded secreted ETEC pathovar-specific antigens: EatA protease, and the EtpA adhesin, as well as the highly conserved chromosomally-encoded YghJ metalloprotease and the EaeH adhesin protein.

**Figure 3:**
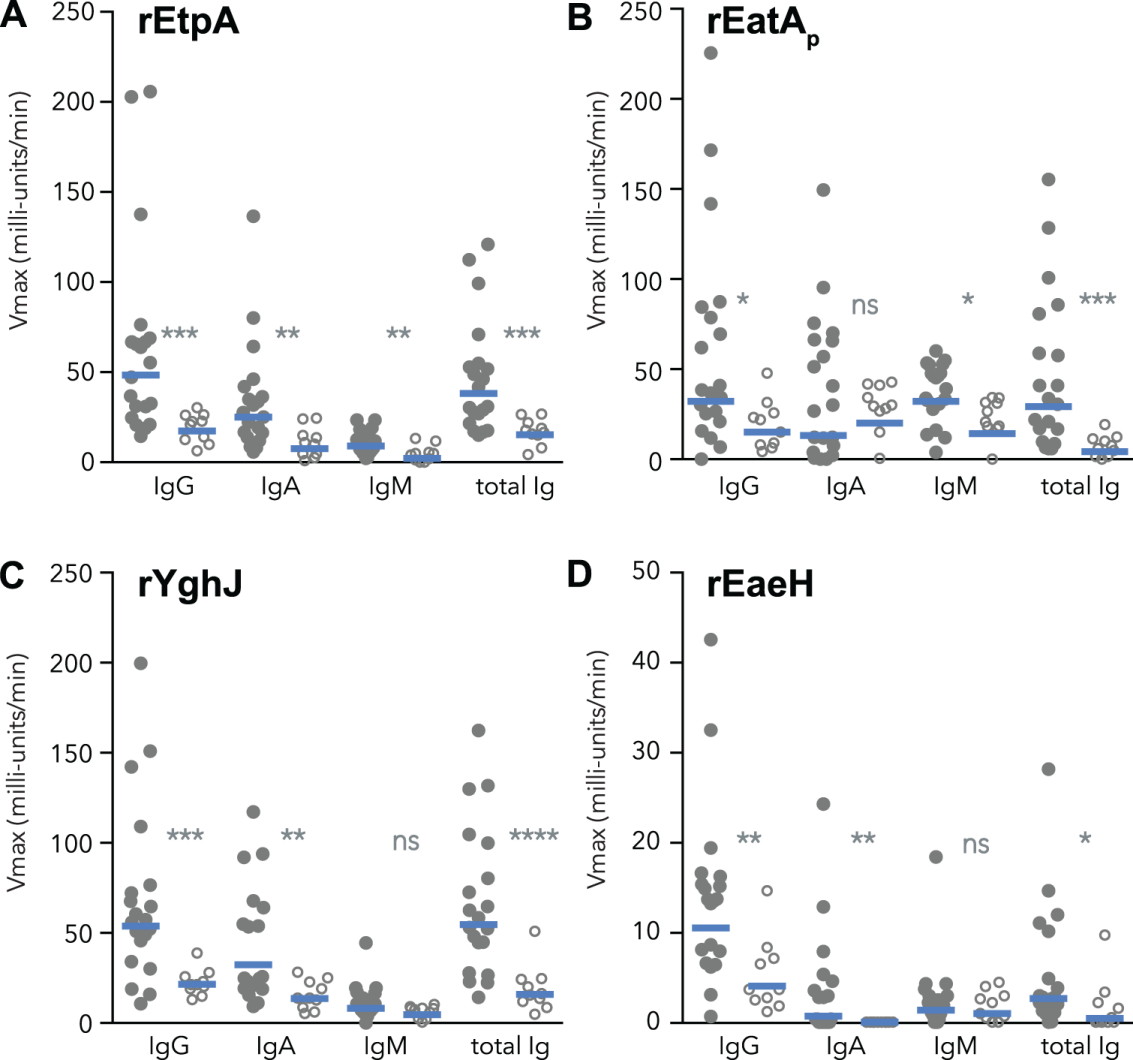
Recognition of novel antigens during naturally occurring ETEC infections in Bangladesh. Shown are kinetic ELISA data for four different recombinant antigens (a, rEtpA; b, the rEatA passenger domain; c, rYghJ; and d, rEaeH) obtained with 1:4096 dilutions of convalescent plasma from ETEC-infected patients hospitalized at ICDDR,B in Dhaka, Bangladesh (closed circles), or control patients not infected with enterotoxigenic *E. coli* (open circles). Horizontal bars represent geometric mean Vmax kinetic ELISA values for each group. P values obtained by two-tailed Mann Whitney testing of groups are summarized (*<0.05; **<0.01; ***<0.001;****<0.0001). x-axis of each graph depicts the specificity of the secondary antibody used in the ELISA (IgG, IgA, IgM, and total IgG, IgA, and IgM).

In comparing convalescent plasma from patients hospitalized at icddr,b to uninfected controls from Bangladesh, we found that patients in general exhibited significantly greater total antibody (IgG, IgM, IgA) responses to each of these antigens following diarrheal illness (Fig. 3) suggesting that these proteins are expressed during the course of infection. Similar results were obtained in comparing plasma from un-infected children from a non-endemic area in the United States (S3 Fig.).

We also examined the immune response to EtpA following infection by examining sera obtained before and after challenge of human volunteers with ETEC H10407. In sera obtained from two independent volunteer challenge studies, we also observed significant increases in immune responses to EtpA (S4 Fig.), strongly suggesting that this secreted protein is specifically recognized following infection by ETEC strains including H10407 that secrete this antigen.

### Protective efficacy of combined EtpA-mutant EatA passenger vaccination

Previous studies have demonstrated that individually, vaccination with either EtpA[31,56] or the passenger domain of the EatA serine protease[29] affords protection against intestinal colonization in mice. The data above suggest that collectively these antigens might significantly extend coverage presently offered by classical approaches to ETEC vaccine development. We therefore questioned whether these two antigens could be successfully combined in a subunit approach. Because we have previously demonstrated that the native secreted EatA passenger domain will degrade intestinal mucin[29] as well as the EtpA adhesin molecule[22], we elected to vaccinate animals with a modified recombinant version of the EatA passenger that lacks protease activity (rEatAp_H134R_).

**Figure 4:**
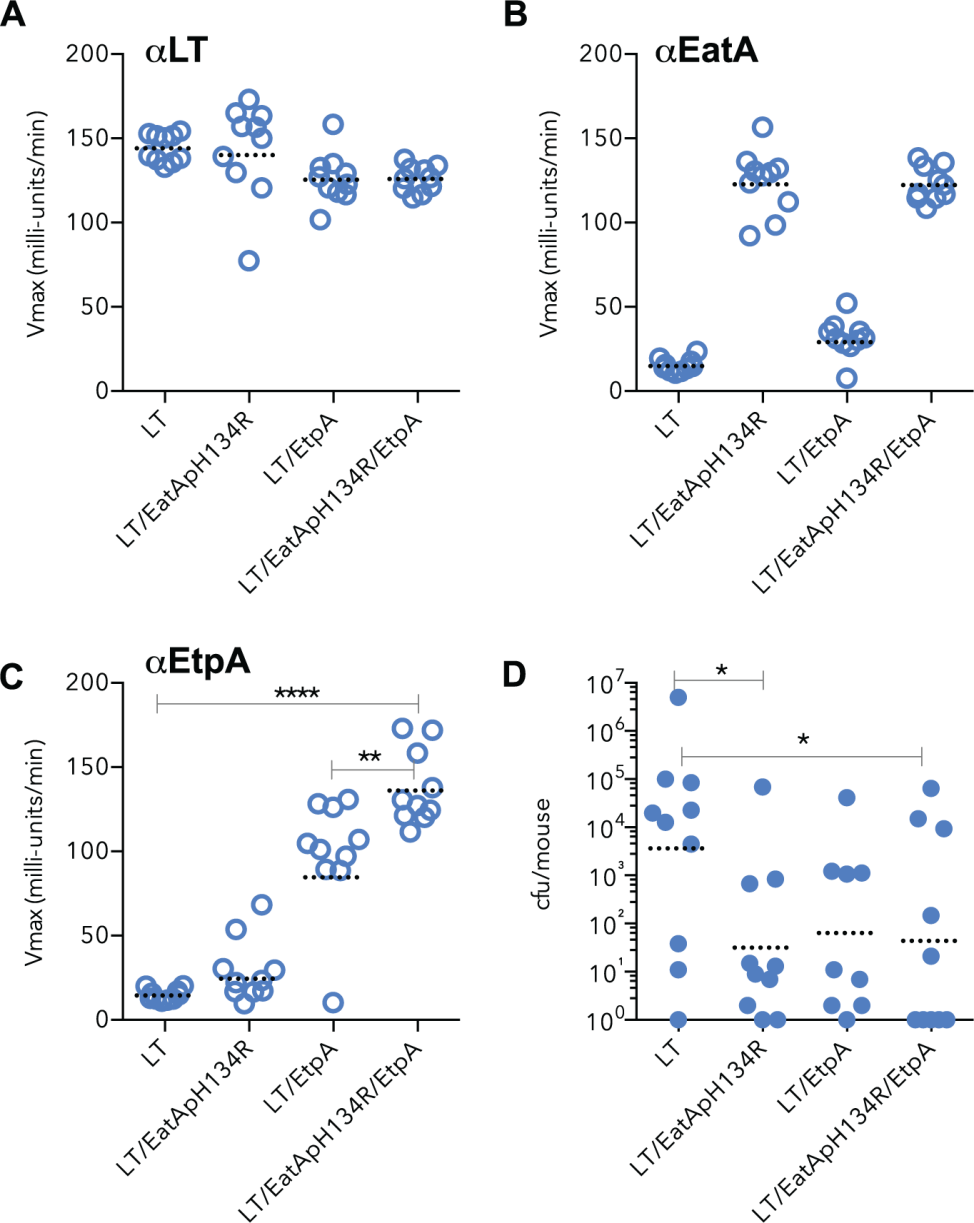
Mice immunized with rEtpA and rEatAp_H134R_ are protected against ETEC infection. Serologic responses to **a** heat-labile toxin (LT), **b** the passenger domain of EatA (Eatp), **c** EtpA. Shown are serum IgG responses following intranasal vaccination of mice with the LT adjuvant alone, or LT with 15 μg of either the proteolytically inactive passenger domain (EatAp_H134R_), EtpA, or both antigens on days 0, 14, 28. Colonization of mice following immunization with the adjuvant alone compared with single and dual antigen vaccination. Comparisons between groups were by Mann Whitney two tailed nonparametric testing. (One mouse died during the vaccination period in the LT/EtpA group and was therefore excluded from the analysis). For mice with no detectable colonies following challenge, the number of cfu is arbitrarily reported as 1 (10°) cfu, the theoretical limit of detection.

Co-vaccination with rEtpA and the mutant rEatAp_H134R_ molecule elicited robust serologic responses to both molecules that were comparable to vaccination with either antigen alone. As anticipated, each of the groups mounted strong serologic responses to the LT adjuvant (Fig. 4A), and both antigens retained their immunogenicity following co-immunization of EtpA with the rEatA_H134R_ passenger domain (Fig. 4B,C) with responses that were at least comparable to those obtained following immunization with either antigen alone (Fig. 4C). Likewise, mice immunized with both antigens were significantly protected against colonization by ETEC (Fig. 4D), although co-vaccination with both antigens did not appear to be more effective than vaccination with either antigen alone. Collectively, however, these data suggest that co-immunization with these two antigens is feasible, and could be employed to expand present approaches to ETEC vaccine antigen selection.

## Discussion

Enterotoxigenic *Escherichia coli* remain one of the most common causes of infectious diarrhea worldwide, and severe disease caused by these pathogens persists as leading cause of death among young children in developing countries[1]. Despite recognition of these toxin producing *E. coli* as a cause of severe cholera-like diarrheal illness more than forty years ago[57], there remains no effective broadly protective vaccine for ETEC.

Most vaccinology efforts to date have focused almost exclusively on a subset of plasmid-encoded antigens, namely the colonization factors (CFs) and heat-labile toxin[9]. Vaccines based on this strategy have faced several impediments. First, the CFs are quite diverse with more than 26 distinct antigens described to date. In addition, a number of recent vaccine studies have suggested that simply engendering immune responses to CFs and/or heat-labile toxin may not be sufficient to provide sustained broad-based protection[14–16].

Recent studies of ETEC pathogenesis suggest that a number of virulence factors in addition to the CFs are involved in efficient delivery of toxins to their cognate receptors on the epithelial surface[2,22,29,30,48,58]. Similarly, the immune response to ETEC infection appears to involve many proteins[25,47] in addition to the classical antigens that are the present focus of most vaccines. Collectively, these findings suggest that there may be additional molecules that could be targeted to interdict toxin delivery by these pathogens, expand the list of potential protective antigens, and complement existing approaches to vaccine development for ETEC[59,60].

A major challenge to ETEC vaccine development in general is that the most highly conserved antigens of ETEC, typically encoded on core regions of the chromosome, are also shared with commensal *E. coli*[60]. Included among these chromosomally encoded conserved proteins are two antigens studied here, YghJ[27] and EaeH[28] that were recently shown to be important for ETEC virulence. While the present studies also demonstrate that these proteins are recognized during the course of ETEC infection, the degree to which these antigens can be safely targeted in vaccines without inadvertent disruption of the intestinal microflora remains to be studied.

The inherent plasticity of *E. coli* genomes contributes substantially to the difficulty in defining antigens unique to the ETEC pathovar that are widely conserved. No single antigen exclusive to these pathogens, but universally conserved in this pathovar, has been described to date. Some have suggested that this might be predicted based on the fact that the plasmid-encoded heat-labile and/or heat-stable toxins, which define the ETEC pathovar, could form a minimal complement of virulence genes in wide variety of *E. coli* host strains[61]. Nevertheless, earlier studies conducted on phlyogenicaly disparate strains from Guinea Bissau[49] and Chile[37] suggested that genes encoding two pathogen-specific antigens EatA and EtpA were present in a majority of strains.

In this context, we examined the gene conservation and the actual production of these proteins in a large collection of well-characterized strains from Bangladesh, complemented by strains from other locations that were associated with severe disease and for which there were available clinical metadata. Notably, two plasmid-encoded ETEC pathotype-specific antigens, the EatA serine protease and the secreted EtpA adhesin molecule were shared broadly among strains belonging to different CF groups with the exception of strains that produced CFA/IV antigens CS4, CS5, CS6 which only infrequently produced EtpA.

The studies reported here represent the largest screen for EtpA and EatA secretion in ETEC performed to date. Earlier studies reporting that genes encoding both proteins were highly conserved relied on either PCR[37] or screening of draft genomes[49] for the presence of the corresponding loci. In general, we found high degrees of concordance between the presence of these genes by PCR and production of the corresponding protein. We should point out however that draft genome assemblies typically fail to encompass the entire *etpA* gene as automated assembly algorithms cannot faithfully incorporate the multiple repeat regions comprising two thirds of *etpA*. This could impact interpretation of gene prevalence in ongoing large-scale ETEC genome sequencing projects. The prevalence of EtpA and EatA (56 and 59%, respectively) as determined by examination of protein expression in our study was slightly lower than previously reported in earlier studies that analyzed strains from Guinea Bissau, where both genes were present in 75% of strains [49]; or Chile, where *etpA* and *eatA*, were present in 71 and 75% of strains, respectively[37]. Nevertheless similar to these earlier studies, the strains that produced these antigens belonged to many different phylogenies suggesting that genes encoding these antigens have been widely dispersed.

The analyses of strains in these studies largely focused on isolates from Bangladesh. However, these data are potentially relevant for vaccine development for a number of reasons. First, Bangladesh is highly endemic for enterotoxigenic *E. coli* infections, and consequently remains an important site for vaccine field trials. In addition, ETEC has been under study in this region since the discovery of this pathotype, permitting us to compare sequence variation in candidate antigens over four decades. Understanding both current prevalence and sequence conservation of potential novel vaccine antigens in this population over time will be particularly important for making rational decisions about their inclusion in future iterations of ETEC vaccines. Finally, the geographic and temporal dispersal of genes encoding EtpA and EatA in multiple phylogenic backgrounds, further attests to importance of studying these molecules as potential vaccine targets as previously suggested by others[37,49].

The optimal formulation of an ETEC vaccine has yet to be defined, and many questions pertaining to the nature of protective immunity that develops following infections with these pathogens remain. Nevertheless, the data presented here do suggest that the novel pathovar-specific antigens could complement existing strategies for ETEC vaccine development by broadening the antigenic valency. Whether the expanded coverage afforded by inclusion of additional pathotype specific antigens would enhance vaccine efficacy beyond that presently achieved by targeting CFs and LT will need to be determined empirically.

## Supporting Information

S1 Fig EatA passenger domain alignments of predicted EatA sequences corresponding to multiple phylogenies from strains belonging to disparate geographic origins. The conserved catalytic triad at amino acids H78, D106, and S211 is highlighted by gray background shading. Geographic origin of strains is depicted in the color key at left of the alignment. Alignments were performed using CLUSTAL Omega (release 1.2.0 AndreaGiacomo) [40] algorithm plugin for CLC Main Workbench.
(PDF)

S2 Fig EtpA alignments of predicted sequences corresponding to multiple phylogenies from strains belonging to disparate geographic origins. Geographic origin of strains is depicted in the color key at left of the alignment. Alignments were performed using sequence alignment algorithm of CLC Main Workbench v6.9.1 with the following parameters: gap open cost = 10.0; gap extension cost = 1.0; end gap cost = as any other; alignment mode = very accurate (slow); redo alignments = no; use fixpoints = no.
(PDF)

S3 Fig Immune response to selected ETEC proteins in infected patients and Bangledeshi and US controls. Shown are (IgG) kinetic ELISA responses (in Vmax, milli-units/min) to recombinant proteins comparing convalescent plasma from patients hospitalized with acute ETEC infections at the International Centre for Diarrhoeal Disease Research in Dhaka, Bangladesh with controls (c) from Bangledeshi adults, and children, as well as plasma from age-matched children from Saint Louis Children’s Hospital (slch). Antigens included two plasmid-encoded ETEC specific antigens (a) EtpA, and (b) the EatA passenger domain; and two chromosomally-encoded conserved antigens (c) YghJ, and (d) EaeH. All plasma samples were diluted 1:4096.
(PDF)

S4 Fig Immune responses to EtpA following volunteer challenge with ETEC H10407. All sera were diluted 1:4096 prior to testing against rEtpA-myc-6His followed by detection of total antibody (IgM,IgG,IgA) in kinetic ELISA. Pre and post values (open and closed circles, respectively) represent collective data from 2 independent ETEC H10407 challenge studies CIR218 and CIR193a. Data from CIR218 are shown as pre-challenge (d-2, open blue circles) and (d28, closed blue circles), while data from CIR193a appear as open grey circles (pre-challenge, d0) and closed grey circles (post challenge, d9). Dashed horizontal lines represent geometric means. P value represents comparison of pre and post-challenge samples by Mann Whitney 2-tailed analysis.
(PDF)

S1 Table Oligonucleotide primers used in this study. Shown in the table are oligonucleotide pairs, and predicted product sizes for ETEC pathotype-specific virulence gene amplification in these studies.
(PDF)

S1 Dataset S1 Dataset.xlsx contains the complete listing of all 181 strains analyzed for production of the secreted EtpA, EatA antigens (tab 1), and YghJ (tab 2). Tab 3 shows 91 unique isolates sequenced by the Genome Sequencing Center for Infectious Diseases (GSCID) which were screened for the presence of the *eaeH* gene by BLASTP homology searches. Note that in the dataset, disease severity is classified numerically (0 = isolate from asymptomatic colonization, 1 = mild disease, 2 = severe cholera-like illness). The presence or absence of a given protein or gene in each strain is defined in a binary fashion where 1 = yes; 0 = no.
(XLSX)

S2 Dataset S2 Dataset.csv contains numerical data corresponding to the heatmap depicted in Fig. 1A. (CSV)
