# Correction: Conservation and Immunogenicity of Novel Antigens in Diverse Isolates of Enterotoxigenic *Escherichia coli*

**DOI:** 10.1371/journal.pntd.0003858

**Published:** 2015-06-15

**Authors:** The PLOS Neglected Tropical Diseases Staff

## Notice of Republication

This article was republished on May 26, 2015, to correct an omission in the Editor section of the article. The Editor of this article was Joseph M. Vinetz. The publisher apologizes for this error. Please download this article again to view the correct version.
