# Supplementary figures and images for: Conservation and Immunogenicity of Novel Antigens in Diverse Isolates of Enterotoxigenic Escherichia coli
Source: PLoS Negl Trop Dis. 2015 Jan 28;9(1):e0003446. doi: 10.1371/journal.pntd.0003446 (PMC4309559; doi:10.1371/journal.pntd.0003446)

## EatA\_passenger\_CLUSTAL\_Omega\_1.2.0

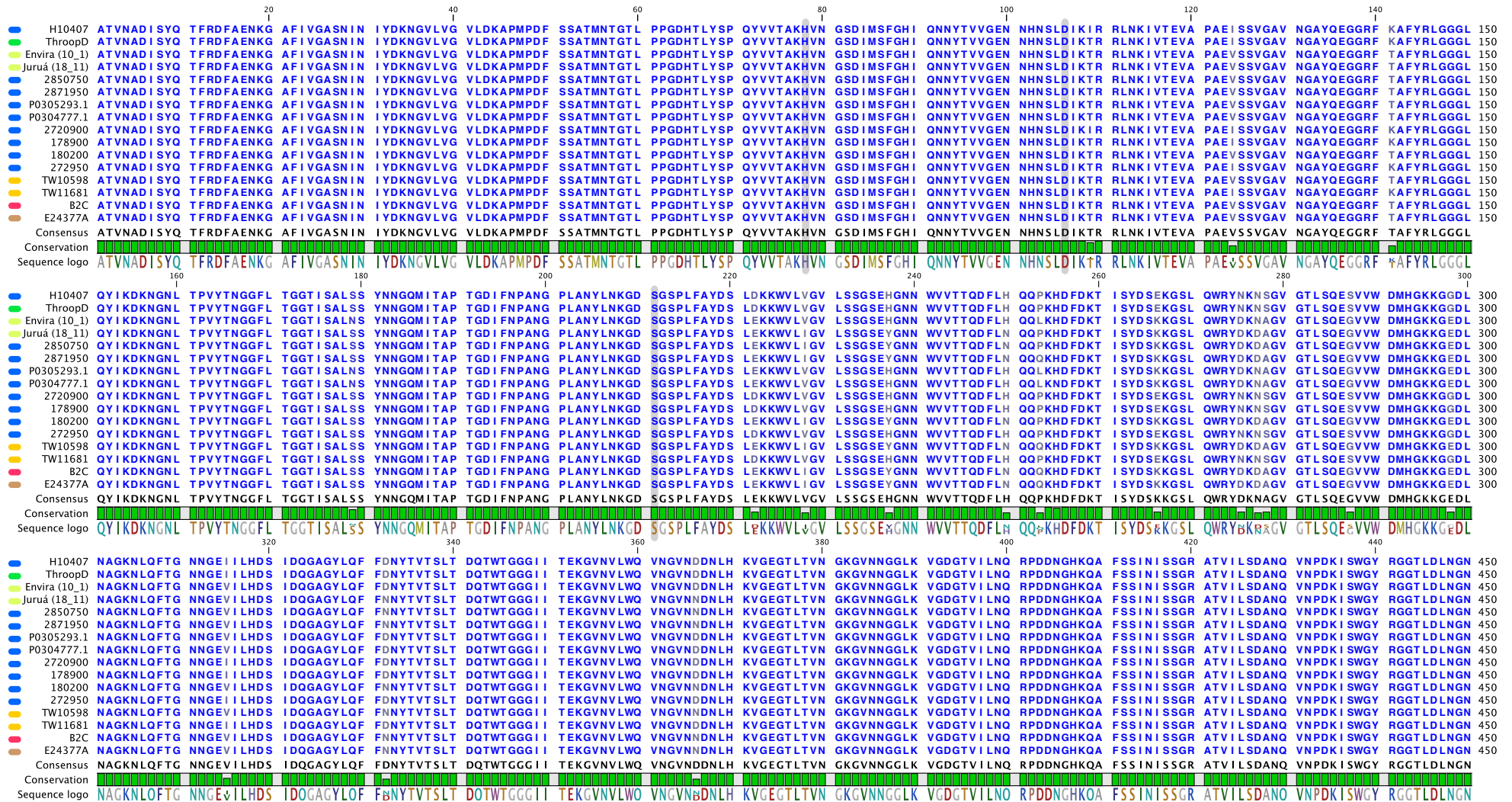

EatA\_passenger\_CLUSTAL\_Omega\_1.2.0

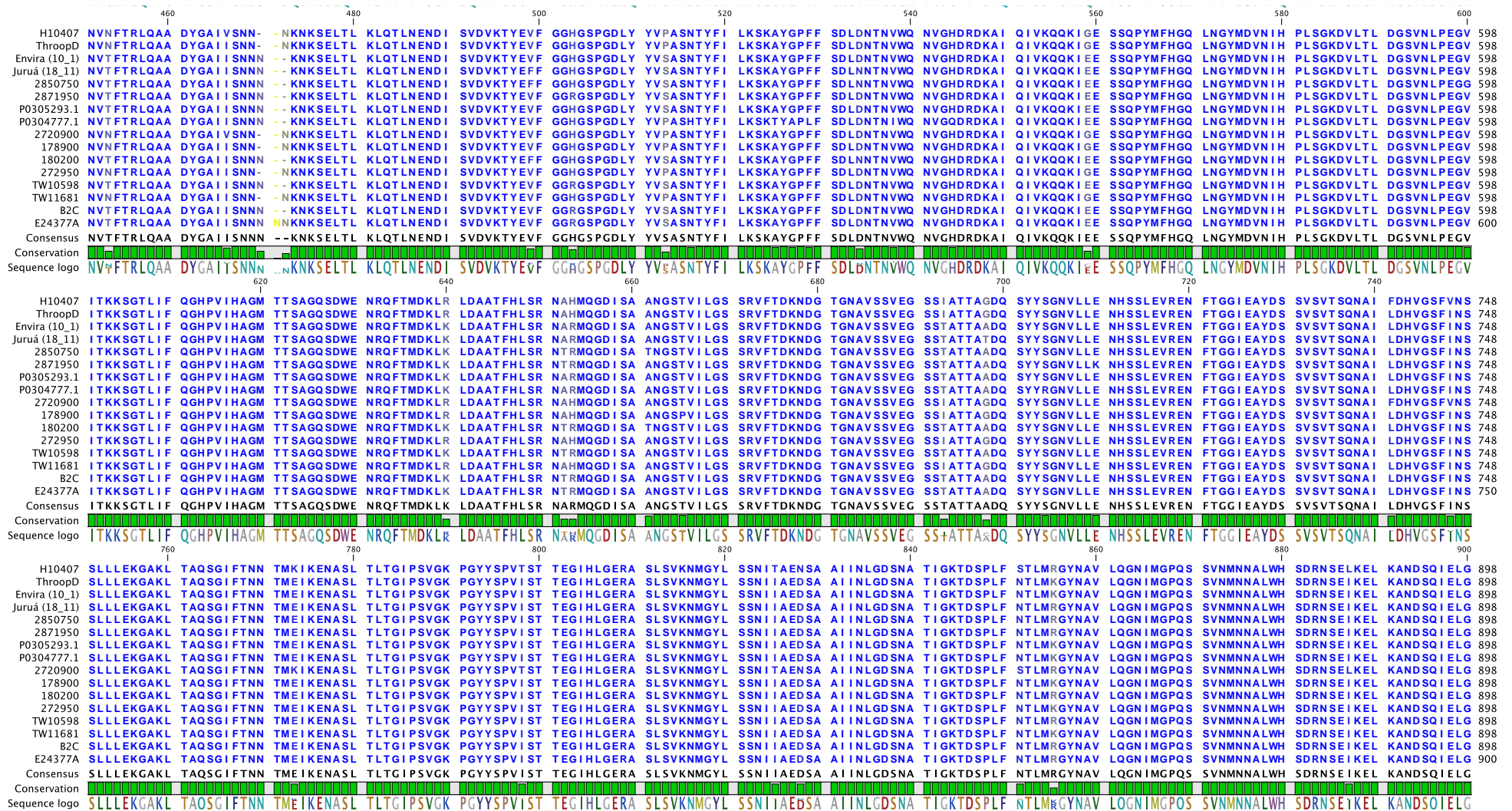

# EatA\_passenger\_CLUSTAL\_Omega\_1.2.0

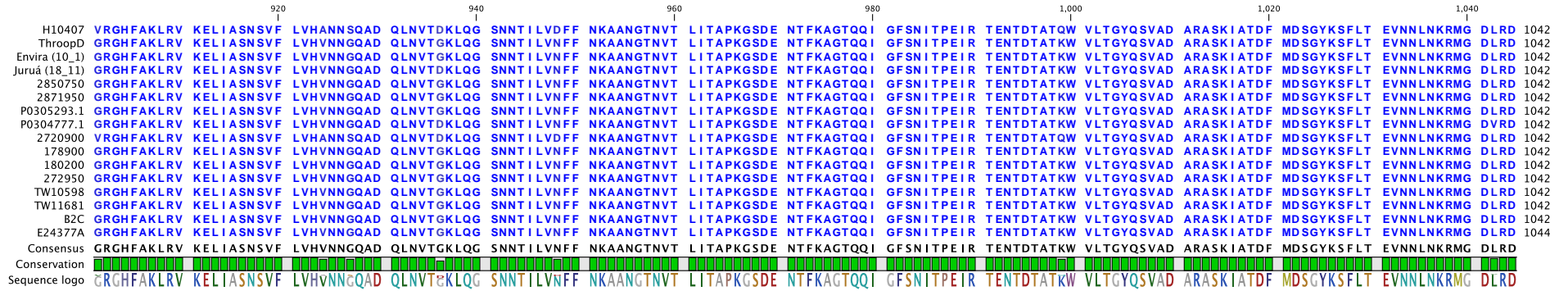

Supplement: S1 Fig — The conserved catalytic triad at amino acids H78, D106, and S211 is highlighted by gray background shading. Geographic origin of strains is depicted in the color key at left of the alignment. Alignments were performed using CLUSTAL Omega (release 1.2.0 AndreaGiacomo) [40] algorithm plugin for CLC Main Workbench. (PDF) [file pntd.0003446.s001.pdf]

•

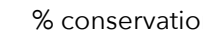

100

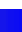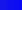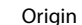

- Bangladesh
- United States
- Amazonia
- Guinea Bissau
- Hong Kong
- Egypt

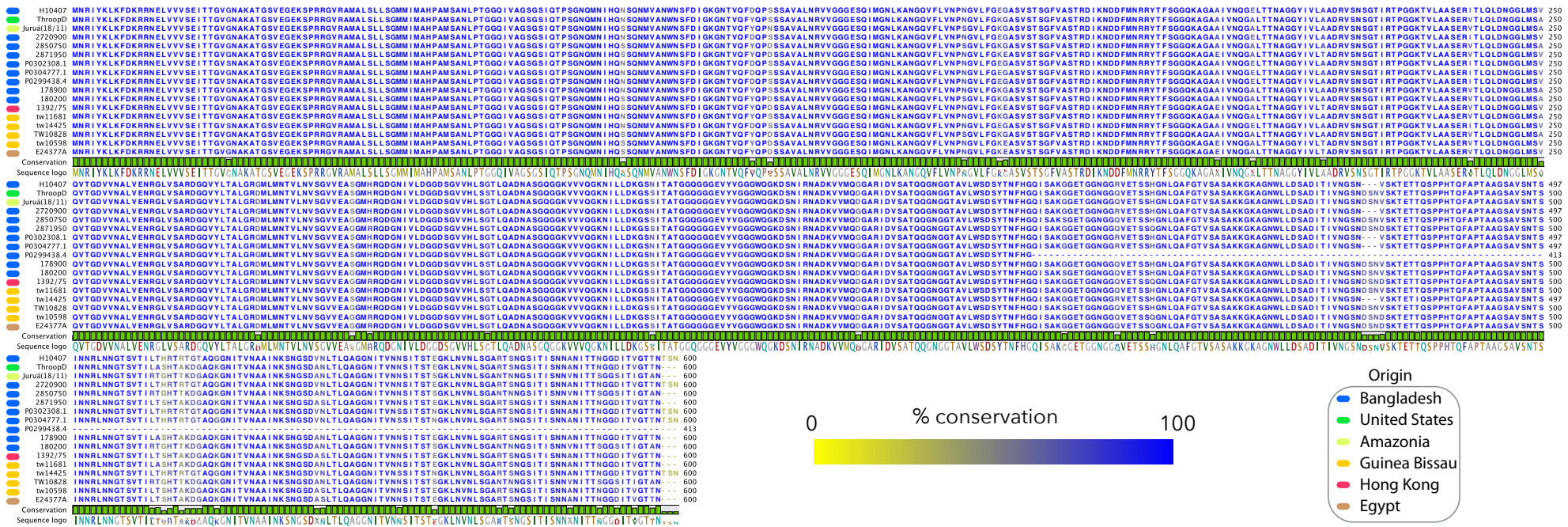

Supplement: S2 Fig — Geographic origin of strains is depicted in the color key at left of the alignment. Alignments were performed using sequence alignment algorithm of CLC Main Workbench v6.9.1 with the following parameters: gap open cost = 10.0; gap extension cost = 1.0; end gap cost = as any other; alignment mode = very accurate (slow); redo alignments = no; use fixpoints = no. (PDF) [file pntd.0003446.s002.pdf]

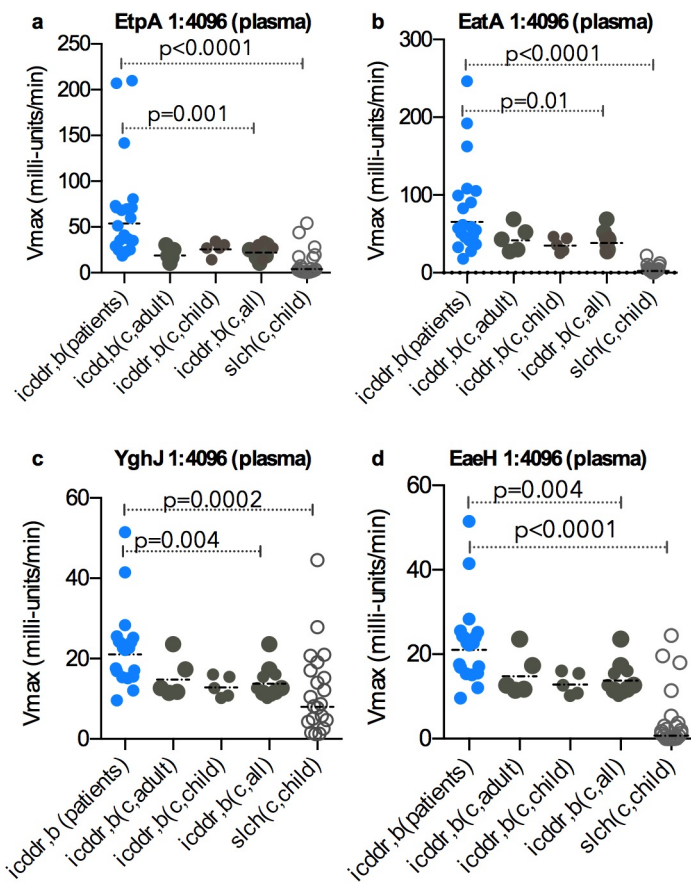

Supplement: S3 Fig — Shown are (IgG) kinetic ELISA responses (in Vmax, milli-units/min) to recombinant proteins comparing convalescent plasma from patients hospitalized with acute ETEC infections at the International Centre for Diarrhoeal Disease Research in Dhaka, Bangladesh with controls (c) from Bangledeshi adults, and children, as well as plasma from age-matched children from Saint Louis Children’s Hospital (slch). Antigens included two plasmid-encoded ETEC specific antigens (a) EtpA, and (b) the EatA passenger domain; and two chromosomally-encoded conserved antigens (c) YghJ, and (d) EaeH. All plasma samples were diluted 1:4096. (PDF) [file pntd.0003446.s003.pdf]
