# Supplementary material for: Conservation and Immunogenicity of Novel Antigens in Diverse Isolates of Enterotoxigenic Escherichia coli
Source: PLoS Negl Trop Dis. 2015 Jan 28;9(1):e0003446. doi: 10.1371/journal.pntd.0003446 (PMC4309559; doi:10.1371/journal.pntd.0003446)
Supplement: S4 Fig — All sera were diluted 1:4096 prior to testing against rEtpA-myc-6His followed by detection of total antibody (IgM,IgG,IgA) in kinetic ELISA. Pre and post values (open and closed circles, respectively) represent collective data from 2 independent ETEC H10407 challenge studies CIR218 and CIR193a. Data from CIR218 are shown as pre-challenge (d-2, open blue circles) and (d28, closed blue circles), while data from CIR193a appear as open grey circles (pre-challenge, d0) and closed grey circles (post challenge, d9). Dashed horizontal lines represent geometric means. P value represents comparison of pre and post-challenge samples by Mann Whitney 2-tailed analysis. (PDF) [file pntd.0003446.s004.pdf]

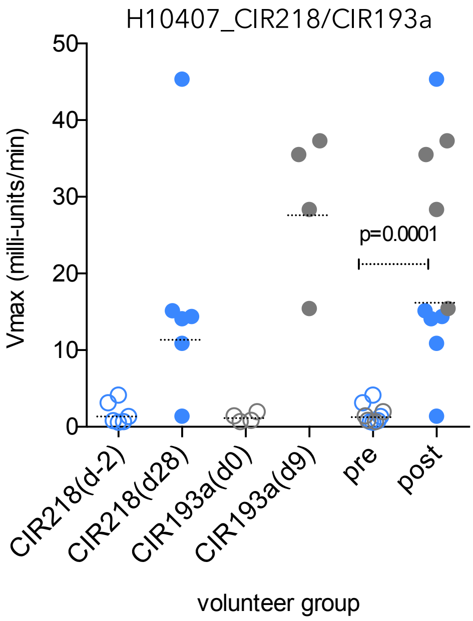

Figure S4. Immune responses to EtpA following volunteer challenge with ETEC H10407.
All sera were diluted 1:4096 prior to testing against rEtpA-myc-6His followed by detection of total antibody (IgM,IgG,IgA) in kinetic ELISA. Pre and post values (open and closed circles, respectively) represent collective data from 2 independent ETEC H10407 challenge studies CIR218 and CIR193a. Data from CIR218 are shown as pre-challenge (d-2, open blue circles) and (d28, closed blue circles), while data from CIR193a appear as open grey circles (pre-challenge, d0) and closed grey circles (post challenge, d9). Dashed horizontal lines represent geometric means. P value represents comparison of pre and post-challenge samples by Mann Whitney 2-tailed analysis.
