# Supplementary material for: Conservation and Immunogenicity of Novel Antigens in Diverse Isolates of Enterotoxigenic Escherichia coli
Source: PLoS Negl Trop Dis. 2015 Jan 28;9(1):e0003446. doi: 10.1371/journal.pntd.0003446 (PMC4309559; doi:10.1371/journal.pntd.0003446)
Supplement: S1 Table — Shown in the table are oligonucleotide pairs, and predicted product sizes for ETEC pathotype-specific virulence gene amplification in these studies. (PDF) [file pntd.0003446.s005.pdf]

supplemental table s1. oligonucleotide primers used in this study

Gene	Primer designation	Sequence (5'>3')	Product size (bp)	Reference
eatA	jf082213.1-F<br>jf082213.2-R	ATGTGCTTTGGCAGGTTAAT<br>ATATCCAGTCAGCACCCACT	1943	[37]
etpA	jf082213.3-F<br>jf082213.4-R	GGTTCAGGCAGTATCCAGAC<br>GGTGTAGCTGTCTGACCACA	999	[37]
eltB	jf092313.3-F<br>jf092313.4-R	ACGGCGTTACTATCCTCTC<br>TGGTCTCGGTCAGATATGTG	273	[34]
est1A	jf092313.5-F<br>jf092313.6-R	TCTTTCCCCTCTTTTAGTCAG<br>ACAGGCAGGATTACAACAAAG	166	[34]
est1B	jf092313.8-F<br>jf092313.7-R	AGTGGTCCTGAAAGCATG<br>TACAAGCAGGATTACAACAC	64	[34]
